# A Theoretical Model of a Simply Supported Circular Ring Under Impulsive Loads

**DOI:** 10.3390/ma19071340

**Published:** 2026-03-27

**Authors:** Yun Xing, Fansen Li, Xin Jia, Yu Yan, Jialing Yang

**Affiliations:** 1 Beijing Advanced Innovation Center for Materials Genome Engineering, Corrosion and Protection Center, Institute for Advanced Materials and Technology, University of Science and Technology Beijing, Beijing 100083, China; m202421614@xs.ustb.edu.cn (F.L.); m202511392@xs.ustb.edu.cn (X.J.); yanyu@ustb.edu.cn (Y.Y.); 2 Institute of Solid Mechanics, School of Aeronautic Science and Engineering, Beihang University, Beijing 100191, China; jlyangbuaa@aliyun.com

**Keywords:** circular ring, rigid plastic, step loading, pulse loading, energy absorption

## Abstract

Metallic thin-walled circular rings are widely employed as energy-absorption components in impact protection systems. However, the dynamic deformation mechanisms under impact loads remain incompletely understood. In this study, we develop a rigid-perfectly plastic model to analyze a simply supported circular ring subjected to impulsive loads. We present a theoretical survey of the incipient deformation under step loading, establishing the relation between the applied load magnitude and the number and location of the stationary plastic hinges. Our analytical findings reveal that as load magnitude increases, the number of stationary hinges grows, with newly formed hinges progressing closer to the point of loading. We validate these theoretical predictions against finite element analyses, demonstrating the model’s accuracy. Additionally, we investigate the complex deformation mechanisms involving both stationary and traveling hinges under rectangular pulse loading. This study provides fundamental insights into the dynamic plastic response of thin-walled structures, offering theoretical guidance for optimizing impact protection systems.

## 1. Introduction

Structures used in transportation, civil, and aerospace engineering are frequently subjected to intense dynamic loads, such as impact and blast loadings [1,2,3]. To mitigate structural damage and improve crashworthiness, a wide range of energy-absorbing structures have been developed, including thin-walled tubes [4], honeycombs [5,6], foam materials [7], and sandwich structures [8]. Among them, metallic circular rings have proven to be excellent energy-absorbing structures due to their stable deformation mode, sufficiently long stroke, high specific energy-absorption capacity, low cost, and easy manufacturing [9]. They have been employed as basic energy-absorbing components in engineering applications, such as the helicopter seats and ship-pile collision protection device [10,11]. Recently, circular rings have been widely adopted as fundamental structural elements in the development of mechanical metamaterials [12,13]. A thorough understanding of the dynamic mechanical behaviors of metallic circular rings is therefore required.

Many studies have been directed toward deciphering the mechanical behaviors of circular rings under quasi-static lateral loading. Based on the rigid-perfectly plastic material model, DeRuntz and Hodge studied the mechanical behaviors of a circular ring under lateral compression load [14]. By considering the material strain hardening effects, the mechanical responses of the lateral compression tubes were investigated [15,16,17]. Based on the slip-line field theory, Sowerby et al. analyzed the collapse load and the four plastic hinge mechanisms of circular rings subjected to diametral concentrated loads [18]. Shim and Stronge investigated the force-displacement response and plastic hinge formation mechanisms of circular tubes subjected to lateral crushing by cylindrical indenters [19]. Karamanos and Andreadakis examined the influence of internal pressure on the local denting behavior of circular tubes under lateral loading [20]. The crushing responses and energy-absorption characteristics of reinforced metal rings have also been investigated through combined experimental and theoretical approaches [21,22]. Beyond single-ring configurations, various modified ring geometries [23,24,25] and nested ring systems [26,27,28] have been proposed to enhance energy-absorption capability under both lateral loading conditions.

In recent years, significant progress has been made in developing advanced theoretical frameworks for the large-deformation and nonlinear analysis of thin-walled structures [29,30,31]. For instance, new analytical approaches for the heavy elastica problem have been proposed to describe large displacements and rotations [32,33]. Engineering theories for thick curved beams have been proposed to predict the complex 3D stress states under both in-plane and out-of-plane loading [34]. Furthermore, Iandiorio and Salvini developed a geometrically nonlinear shell theory for thin-walled tubes and beams subjected to large displacements and cross-section deformation, which effectively captures both section deformation effects and the transition to softening behavior in bending [35].

However, while these models provide high accuracy descriptions of geometric nonlinearities and large deformations, they are primarily established within a static or quasi-static framework. The dynamic response of thin-walled structures under impulsive loading presents a different challenge, where the rapid initiation and evolution of plastic hinge mechanisms dominate the early stage of deformation. In such cases, the classical rigid-perfectly plastic and small-deflection framework remains a powerful and mathematically consistent tool for clarifying the relation between load magnitude and the formation of plastic hinges. Hashmi et al. employed numerical and energy methods to investigate the plastic deformation mechanisms of single rings of different materials under impact loading [36]. Wang et al. proposed an analytical model to investigate the dynamic behaviors of circular rings subjected to wedge-type impact and analyzed the effects of strain rate and strain hardening [37]. Further extending the investigation to the system level, researchers have explored nested structural configurations. The energy absorption capability of nested circular rings under lateral impact was investigated experimentally and numerically, and a design strategy incorporating cylindrical dampers was proposed to achieve a more desirable force–displacement response [38,39]. Furthermore, Wang et al. developed an analytical model to study the dynamic responses of nested circular ring systems under lateral impact and successfully predicted their staged crushing behaviors and stepwise force-time history [40].

Although there are many investigations on the plastic deformations of circular rings [41,42,43,44,45], there are few studies on the complete solution for circular rings subjected to dynamic load. The velocity field of the complete solution should satisfy the equation of motion, equation of compatibility, boundary conditions, initial conditions, and yield criterion throughout the structure during the whole dynamic response. Although the complete solution is mathematically involved, it provides a more accurate and physically consistent description of the plastic deformation and mechanical response of circular rings under impact [46]. For instance, Stronge et al. conducted the complete solutions for a circular ring supported by a pinned connection under a suddenly applied load and presented the four-hinge and five-hinge modes [47]. As the load further increases, a new hinge emerges at the pinned support and makes the deformation mode more complicated. In this configuration, the circular ring can be regarded as being simply supported at its base. However, a rigorous complete solution for this case has not been fully established. In recent years, mechanical metamaterials based on circular ring unit cells have attracted increasing attention owing to their broad potential in impact protection [48]. A systematic investigation of the impact response of simply supported circular rings is of significant scientific interest and practical engineering relevance.

In this paper, a rigid-–perfectly plastic analytical model is developed for the incipient deformation mechanisms of a simply supported circular ring subjected to impulsive loading. The theoretical solutions for the incipient deformation mechanism under step loading are systematically explored over different ranges of load magnitude, and the evolution of the number and location of stationary plastic hinges are clarified. The theoretical predictions are validated through finite element simulations. Furthermore, the deformation mechanisms involving both stationary and traveling plastic hinges under rectangular pulse loading are examined. By providing clear physical insights into the incipient initiation and evolution of plastic hinges, this study establishes a foundation for subsequent nonlinear and large-deformation analyses, thereby offering a general understanding of the plastic deformation mechanisms in circular ring systems under more complicated impact loads and boundary constraints.

## 2. Analytical Model for Step Loading

**Figure 1:**
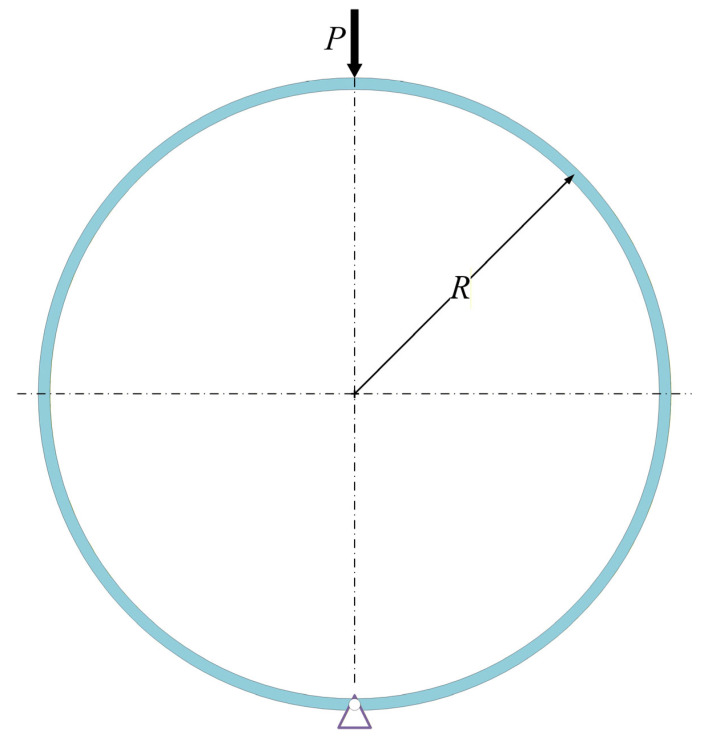
Sketch of a circular ring subjected to concentrated step loading.

**Figure 2:**
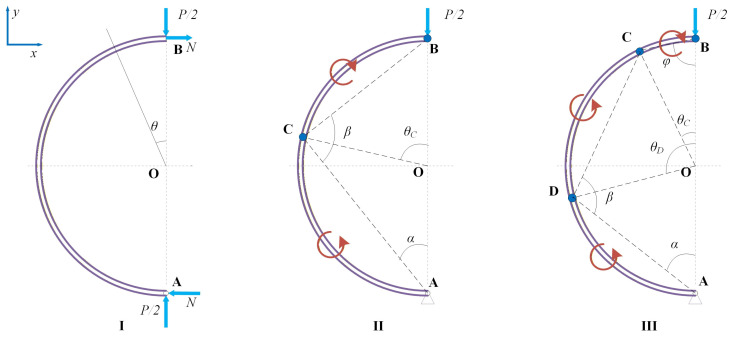
Different deformation modes of a circular ring under step loading.

Figure 1 shows a small-curvature circular ring with uniform radius *R*, thickness *h*, and width *b*, which is simply supported at its bottom. This boundary condition is representative of a possible engineering configuration when circular rings are used as energy-absorbing components. More importantly, the symmetric deformation under simply supported conditions allows the fundamental evolution of plastic hinges to be examined in a relatively clear manner. In this case, the present configuration provides a basic framework for understanding the more complicated constraints. A compressive step loading *P* along the vertical diameter is applied on the top of the circular ring. The yield stress of material is denoted as $\sigma_{s}$. The circular ring may undergo elastic or plastic deformation, which is dependent on the magnitude of the externally applied load. In this study, the following assumptions are made: (1) the material is rigid-perfectly plastic and rate-independent; (2) the deflection of the circular ring is small and only the initial response is considered; and (3) the ratio of thickness to radius of the circular ring is small, and the effect of axial force and shear force on the yielding criterion of the circular ring is neglected [49,50,51]. As *P* increases, the deformation modes of the circular ring are given in Figure 2. Due to symmetry, only half of the deformation is plotted, in which *N* represents the axial force.

### 2.1. Regime I: Static Mode

As *P* is quite small, the circular ring undergoes elastic deformation. From the theory of mechanics of materials [52], the bending moment at arbitrary cross-section *θ* can be written as

(1) $$M(\theta )=-\frac{P}{2}R\sin\theta +\frac{2}{3\pi }PR(1+\cos\theta ).$$

As dM(θ)/dθ=0, one can easily get the extreme point $\theta_{0}=\arctan(-3\pi /4)$. Substituting $\theta_{0}$ into Equation (1), one has

(2) $$M(\theta_{0})=\frac{4-\sqrt{9\pi^{2}+16}}{6\pi }PR.$$

From Equation (1), the bending moment at point B can be expressed as M(0)=4PR/(3π). Apparently, $\left|M(\theta_{0})\right|<\left|M(0)\right|$; that is, a plastic hinge may form first at point B as $\left|M(0)\right|=M_{p}$, where *M_p_* is the fully plastic bending moment and $M_{p}=\sigma_{s}bh^{2}/4$. The plastic limit load *P*_1_ when $\left|M(0)\right|=M_{p}$ is

(3) $$\frac{P_{1}R}{M_{p}}=\frac{3\pi }{4}.$$

Introduce a non-dimensional parameter $\bar{P}=PR/M_{p}$, then ${\bar{P}}_{1}=3\pi /4$. As $\bar{P}\geq {\bar{P}}_{1}$, a plastic hinge forms at point B. Considering the rigid-perfectly plastic assumption, the bending moment at point B is always *M_p_*. The axial force at A is $N_{A}=M_{p}/(2R)$. From the equilibrium equation of moment, one has

(4) $$M(\theta )=-\frac{P}{2}R\sin\theta +\frac{M_{p}}{2}(1+\cos\theta ).$$

From the condition dM(θ)/dθ=0, the extreme point is located at

(5) $$\theta_{p}=\arctan(\frac{-PR}{M_{p}})=\arctan(-\bar{P}),$$

and the corresponding peak value of the bending moment is

(6) $$M(\theta_{p})=\frac{M_{p}}{2}(1-\sqrt{1+{\bar{P}}^{2}}).$$

From the above two equations, it is clear that the extreme point moves towards point B as $\bar{P}$ increases. If $\bar{P}=2\sqrt{2}$, $\left|M(\theta_{p})\right|$ will equal *M_p_*, which means that a new plastic hinge C forms at $\theta =\theta_{C}=\arctan(-2\sqrt{2})+\pi$. Due to symmetry, there is another plastic hinge at the symmetric location on the right half of the circular ring; i.e., there will be three plastic hinges in the circular ring. As long as $\bar{P}<{\bar{P}}_{2}=2\sqrt{2}$, the circular ring remains static, and the single-hinge mode is valid as ${\bar{P}}_{1}\leq \bar{P}<{\bar{P}}_{2}=2\sqrt{2}$.

### 2.2. Regime II: Three-Hinge Mechanism

When three plastic hinges exist in the circular ring, the circular ring becomes a mechanism. With the increase in *P*, the plastic hinge C continues to move. There is only one degree of freedom. As shown in Figure 2, (*x*, *y*) is the global coordinate system, and *u*, *v* are the velocity components along the *x* and *y* coordinates, respectively. The segment AC rotates in a counterclockwise direction around point A. The segment BC rotates clockwise, and point B moves along the negative *y* axis. At the initial instant, the geometric relation is

(7) $$\beta =\left(\alpha -\alpha_{0}\right)\cot^{2}\frac{\theta_{C}}{2}+\beta_{0},$$

where *β* and *α* are the angles between line BC and AC, and AC and AB, respectively. *β*_0_ and *α*_0_ are their initial values. The geometrical relation can be obtained by

(8) $$\dot{\beta }=-\dot{\alpha }\csc^{2}\frac{\theta_{C}}{2},$$

where the dot denotes the derivative with respect to time. The velocity at point C is

(9) $$u_{C}=-2R\dot{\alpha }\cos^{2}\frac{\theta_{C}}{2},$$

and

(10) $$v_{C}=-R\dot{\alpha }\sin\theta_{C}.$$

The velocity at the arbitrary position of the circular ring is

(11) $$\left\{\begin{array}{l} u(\theta )=u_{C}+\left(\sin\theta -\sin\theta_{C}\right)R\left(\dot{\alpha }+\dot{\beta }\right)\\ v(\theta )=v_{C}+\left(\cos\theta -\cos\theta_{C}\right)R\left(\dot{\alpha }+\dot{\beta }\right) \end{array}\right.\;\mathrm{for}\;0\leq \theta <\theta_{C},$$

and

(12) $$\left\{\begin{array}{l} u(\theta )=-R\dot{\alpha }(1+\cos\theta )\\ v(\theta )=-R\dot{\alpha }\;\sin\theta \end{array}\right.\;\mathrm{for}\;\theta_{C}\leq \theta <\pi .$$

As the bending moment gets the extreme value, the shear force equals zero, and only the axial force *N_C_* exists. For segment BC, the momentum in the *y* direction is

(13) $$\begin{array}{ll} I_{BC} & =\rho Rbh\int_{0}^{\theta_{C}}v\left(\theta \right)d\theta \\ & =\rho bhR^{2}\left[\left(-\sin\theta_{C}\right)\theta_{C}+\left(\cos\theta_{C}+\theta_{C}\sin\theta_{C}-1\right)\left(-\cot^{2}\frac{\theta_{C}}{2}\right)\right]\dot{\alpha } \end{array}.$$

The translational equation of motion in the *y* direction is

(14) $$N_{C}\sin\theta_{C}-\frac{P}{2}=\frac{d}{dt}I_{BC}.$$

Substituting Equation (13) into (14), one has

(15) $$N_{C}\sin\theta_{C}-\frac{P}{2}=\rho bhR^{2}\left[\left(-\sin\theta_{C}\right)\theta_{C}+\left(\sin\theta_{C}-\theta_{C}\cos\theta_{C}\right)\left(-\cot^{2}\frac{\theta_{C}}{2}\right)\right]\ddot{\alpha }.$$

For segment BC, the angular momentum about point B is

(16) $$\begin{array}{ll} J_{BC} & =\rho Rbh{\int_{0}^{\theta_{C}}\dot{\gamma }\left(2R\sin\frac{\theta }{2}\right)}^{2}d\theta +\rho Rbh\int_{0}^{\theta_{C}}{\dot{v}}_{B}R\sin\theta d\theta \\ & =2\rho bhR^{3}\left[\cot^{2}\frac{\theta_{C}}{2}\left(\theta_{C}-\sin\theta_{C}\right)-\cot\frac{\theta_{C}}{2}\rho R^{2}\left(1-\cos\theta \right)\right]\dot{\alpha } \end{array}.$$

The rotational equation of motion is

(17) $$2N_{C}R\sin^{2}\frac{\theta_{C}}{2}-2M_{p}=\frac{d}{dt}J_{BC}.$$

Substitution of Equation (16) into (17) gives

(18) $$2N_{C}R\sin^{2}\frac{\theta_{C}}{2}-2M_{p}=2\rho bhR^{3}\cot^{2}\frac{\theta_{C}}{2}\left(\theta_{C}-\sin\theta_{C}\right)\ddot{\alpha }.$$

For segment AC, the angular momentum about point A is

(19) $$J_{AC}=\rho Rbh{\int_{\theta_{C}}^{\pi }\dot{\alpha }\left(2R\cos\frac{\theta }{2}\right)}^{2}d\theta =2\rho bhR^{3}\left(\pi -\theta_{C}-\sin\theta_{C}\right)\dot{\alpha }$$

The rotational equation of motion is

(20) $$2N_{C}R\cos^{2}\frac{\theta_{C}}{2}-M_{p}=\frac{d}{dt}J_{AC}.$$

By substituting Equation (19) into Equation (20), one gets

(21) $$2N_{C}R\cos^{2}\frac{\theta_{C}}{2}-M_{p}=2\rho bhR^{3}\left(\pi -\theta_{C}-\sin\theta_{C}\right)\ddot{\alpha }.$$

Combining Equations (15), (18), and (21), *θ_C_*, $\ddot{\alpha }$, and *F_C_* can be solved as *P* is given. Then the bending moment for segments BC and AC are, respectively,

(22) $$\begin{array}{ll} M\left(\theta \right) & =N_{C}R\left[1-\cos\left(\theta_{C}-\theta \right)\right]-M_{p}\\ & \;+\rho Rbh\int_{\theta }^{\theta_{C}}\dot{v}\left(\xi \right)R\left(\sin\theta -\sin\xi \right)d\xi \\ & \;+\rho Rbh\int_{\theta }^{\theta_{C}}\dot{u}(\xi )R\left(\cos\theta -\cos\xi \right)\; \end{array},$$

and

(23) $$\begin{array}{ll} M\left(\theta \right) & =N_{C}R\left[1-\cos\left(\theta_{C}-\theta \right)\right]-M_{p}\\ & \;-\rho Rbh\int_{\theta_{C}}^{\theta }\left(2R\ddot{\alpha }\cos\frac{\xi }{2}\right)\left(2R\sin\frac{\theta }{2}\right)\sin\left(\frac{\theta }{2}-\frac{\xi }{2}\right)d\xi \end{array}.$$

### 2.3. Regime III: Five-Hinge Mechanism

When the step loading *P* increases further, the bending moment in segment AC increases according to Equation (22). Another plastic hinge D will appear on the left half circular ring, and the whole ring will deform as a mechanism with five plastic hinges. As shown in Figure 2, regime III of the circular ring has two degrees of freedom. The segments AD and DC rotate counterclockwise, and segment BC rotates clockwise. Point B still moves along the negative *y* axis. Denote *α* and *β* as the two generalized coordinates. The velocity at point D is

(24) $$u_{D}=-2R\dot{\alpha }\cos^{2}\frac{\theta_{D}}{2},$$

and

(25) $$v_{D}=-R\dot{\alpha }\sin\theta_{D}.$$

The velocity at point C is

(26) $$u_{C}=u_{D}-2R\sin\left(\frac{\theta_{D}}{2}-\frac{\theta_{C}}{2}\right)\sin\left(\frac{\theta_{D}}{2}+\frac{\theta_{C}}{2}\right)\left(\dot{\alpha }+\dot{\beta }\right),$$

and

(27) $$v_{C}=v_{D}+2R\sin\left(\frac{\theta_{D}}{2}-\frac{\theta_{C}}{2}\right)\cos\left(\frac{\theta_{D}}{2}+\frac{\theta_{C}}{2}\right)\left(\dot{\alpha }+\dot{\beta }\right).$$

Due to symmetry, the horizontal velocity at point B satisfies $u_{B}=0$. From the geometric relation, we have

(28) $$v_{B}=v_{C}+u_{C}\cot\frac{\theta_{C}}{2}.$$

The velocities for segments BC, CD, and DA are

(29) $$\left\{\begin{array}{l} u\left(\theta \right)=\sin^{2}\frac{\theta }{2}\csc^{2}\frac{\theta_{C}}{2}u_{C}\\ v\left(\theta \right)=v_{C}+u_{C}\cot\frac{\theta_{C}}{2}+\frac{1}{2}\sin\theta \left[-\csc^{2}\frac{\theta_{C}}{2}u_{C}\right] \end{array}\right.\;\mathrm{for}\;0\leq \theta <\theta_{C},$$

(30) $$\left\{\begin{array}{c} u\left(\theta \right)=u_{D}-\left(\cos\theta -\cos\theta_{D}\right)R\left(\dot{\alpha }+\dot{\beta }\right)\\ v\left(\theta \right)=v_{D}+\left(\sin\theta -\sin\theta_{D}\right)R\left(\dot{\alpha }+\dot{\beta }\right) \end{array}\right.\;\mathrm{for}\;\theta_{C}\leq \theta <\theta_{D},$$

and

(31) $$\left\{\begin{array}{l} u\left(\theta \right)=-R\dot{\alpha }\left(\cos\theta +1\right)\\ v\left(\theta \right)=-R\dot{\alpha }\sin\theta \end{array}\right.\;\mathrm{for}\;\theta_{D}\leq \theta <\pi ,$$

respectively, where

(32) $$\varphi =\varphi_{0}+\frac{1}{2R}\left(\cot^{2}\frac{\theta_{C}}{2}+1\right)\left[2R\cos^{2}\left(\frac{\theta_{D}}{2}\right)\alpha +2R\sin\left(\frac{\theta_{D}}{2}-\frac{\theta_{C}}{2}\right)\sin\left(\frac{\theta_{D}}{2}+\frac{\theta_{C}}{2}\right)\left(\alpha +\beta \right)\right],$$

*φ* is the angle between the tangent of segment CB and line OB, and *φ*_0_ is the initial value.

The translational equations of motion in the *x* and *y* direction for segment BC are

(33) $$N_{B}-N_{C}\cos\theta_{C}=\rho Rbh\int_{0}^{\theta_{C}}\dot{u}d\theta ,$$

and

(34) $$-N_{C}\sin\theta_{C}-\frac{P}{2}=\rho Rbh\int_{0}^{\theta_{C}}\dot{v}d\theta ,$$

respectively.

The rotational equation of motion of segment BC about B is

(35) $$\begin{array}{ll} -M_{C}-M_{B}-N_{C}R\left(1-\cos\theta_{C}\right) & =\rho Rbh\int_{0}^{\theta_{C}}-\dot{u}R\left(1-\cos\theta \right)d\theta \\ & \;+\rho Rbh\int_{0}^{\theta_{C}}\dot{v}R\sin\left(\theta \right)d\theta \end{array},$$

where $M_{B}$ and $M_{C}$ are the bending moments at plastic hinges B and C, respectively. The translational equations of motion in the *x* and *y* directions for segment CD are

(36) $$N_{C}\cos\theta_{C}-N_{D}\cos\theta_{D}=\rho Rbh\int_{\theta_{C}}^{\theta_{D}}\dot{u}d\theta ,$$

and

(37) $$N_{C}\sin\theta_{C}-N_{D}\sin\theta_{D}=\rho Rbh\int_{\theta_{C}}^{\theta_{D}}\dot{v}d\theta ,$$

respectively.

The rotational equation of motion of segment CD about C is

(38) $$\begin{array}{ll} M_{D}+M_{C}-N_{D}R\left(1-\cos\left(\theta_{D}-\theta_{C}\right)\right) & =\rho Rbh\int_{\theta_{C}}^{\theta_{D}}-\dot{u}\left(\cos\theta_{C}-\cos\theta \right)d\theta \\ & \;+\rho Rbh\int_{\theta_{C}}^{\theta_{D}}\dot{v}\left(\sin\theta -\sin\theta_{C}\right)d\theta \end{array},$$

where $M_{D}$ is the bending moment at plastic hinges D. The rotational equation of motion for segment AD is

(39) $$-M_{D}+2N_{D}R\cos^{2}\frac{\theta_{D}}{2}=\rho Rbh\int_{\theta_{D}}^{\pi }\left(\dot{v}\sin\theta \right)Rd\theta +\rho Rbh\int_{\theta_{D}}^{\pi }\dot{u}\left(1+\cos\theta \right)Rd\theta .$$

In this section, effects of shear and axial forces on yielding are neglected. The bending moments at plastic hinges B, C, and D satisfy $M_{B}=M_{C}=M_{D}=M_{p}$. By using Equations (34)–(39), *N_C_*, *N_D_*, *θ_C_*, *θ_D_*, $\ddot{\alpha }$, and $\ddot{\beta }$ can be solved. Then the bending moments for segments BC, CD, and DA are

(40) $$\begin{array}{l} M\left(\theta \right)=F_{C}R\left[1-\cos\left(\theta_{C}-\theta \right)\right]-M_{p}+\rho Rbh\int_{\theta }^{\theta_{c}}\dot{v}\left(\xi \right)R\left(\sin\theta -\sin\xi \right)d\xi \\ \;\;\;\;\;\;\;\;\;\;\;+\rho Rbh\int_{\theta }^{\theta_{C}}-\dot{u}\left(\xi \right)R\left(\cos\xi -\cos\theta \right)d\xi \end{array},$$

(41) $$\begin{array}{l} M\left(\theta \right)=F_{D}R\left[1-\cos\left(\theta_{C}-\theta \right)\right]+M_{p}+\rho Rbh\int_{\theta }^{\theta_{D}}\dot{v}\left(\xi \right)R\left(\sin\theta -\sin\xi \right)d\xi \\ \;\;\;\;\;\;\;\;\;\;\;+\rho Rbh\int_{\theta }^{\theta_{D}}-\dot{u}\left(\xi \right)R\left(\cos\xi -\cos\theta \right)d\xi \end{array},$$

and

(42) $$\begin{array}{l} M\left(\theta \right)=-F_{D}R\left[1-\cos\left(\theta_{D}-\theta \right)\right]+M_{p}-\rho Rbh\int_{\theta }^{\theta_{C}}\dot{v}\left(\xi \right)R\left(\sin\theta -\sin\xi \right)d\xi \\ \;\;\;\;\;\;\;\;\;\;\;-\rho Rbh\int_{\theta }^{\theta_{C}}-\dot{u}\left(\xi \right)R\left(\cos\xi -\cos\theta \right)d\xi \end{array},$$

respectively.

## 3. Analytical Model for Pulse Loading

Consider a compressive pulse loading *P* with constant magnitude *P*_0_ and duration *t*_1_ acting on the top of the simply supported circular ring along the vertical diameter. *P* is described as

(43) $$P=\left\{\begin{array}{cc} P_{0}, & 0\leq t<t_{1}\\ 0, & t_{1}\leq t \end{array}\right..$$

Under the same load magnitude, when $t<t_{1}$, the deformation mechanism of the circular ring under pulse loading is the same as that of step loading, as shown in Figure 2. As the number of the plastic hinges increases, it is difficult to get an analytical solution for the pulse loading. Thus, only the three-hinge mechanism in Figure 2 is discussed for pulse loading. The model is restricted to small and moderate impact loads. For higher pulse loads where five-hinge or higher-order mechanisms are activated, this specific analytical model may be replaced by numerical methods, such as the finite element approaches validated in Section 4.2.

### 3.1. Phase I

From Figure 2, it is known that the first plastic hinge B is located at the loading position. The second hinge C is located at $\theta =\theta_{1}$, and the third hinge is located at the symmetrical position of hinge C. Under the applied load *P*_0_, the rigid segments BC and AC connected by hinge C rotate with a uniform acceleration. By setting *P* = *P*_0_, the angular coordinate *θ*_1_ and angular acceleration ${\ddot{\alpha }}_{0}$ of segment AC can be solved by utilizing Equations (15), (18), and (21).

In this phase, the plastic hinges C and B are both stationary hinges. The input energy is transformed into the kinetic energy of the rigid segments and the plastic energy in the stationary hinges. When this phase ends at $t=t_{1}$, the angular velocity and angular displacement of segment AC are

(44) $${\dot{\alpha }}_{1}={\ddot{\alpha }}_{0}t_{1},$$

and

(45) $$\alpha_{1}=\frac{1}{2}{\ddot{\alpha }}_{0}t_{1}^{2}.$$

### 3.2. Phase II

When $t\geq t_{1}$, the applied load *P*_0_ is removed and the stationary-hinge mechanism of Phase I is unsustainable. The plastic hinge C becomes a traveling hinge and moves along the circular ring. The plastic hinge B is still stationary. Due to the movement of the plastic hinge C, the translational equation of motion of segment BC in the *y* direction is modified as

(46) $$F_{C}\sin\theta_{C}=\frac{dI_{BC}}{dt}=\frac{dI_{BC}}{d\dot{\alpha }}\ddot{\alpha }+\frac{dI_{BC}}{d\theta_{C}}{\dot{\theta }}_{C}.$$

By substituting Equation (13) into Equation (46), one gets

(47) $$\begin{array}{l} F_{C}\sin\theta_{C}\\ =\rho R^{2}\left[\left(-\sin\theta_{C}\right)\theta_{C}+\left(\cos\theta_{C}+\theta_{C}\sin\theta_{C}-1\right)\left(-\cot^{2}\frac{\theta_{C}}{2}\right)\right]\ddot{\alpha }\\ +\rho R^{2}\left[\left(-\sin\theta_{C}\right)+\left(-\cos\theta_{C}\right)\theta_{C}+Q+\left(\theta_{C}\cos\theta_{C}\right)\left(-\cot^{2}\frac{\theta_{C}}{2}\right)\right]\dot{\alpha }{\dot{\theta }}_{C} \end{array},$$

where $Q=\left(\cos\theta_{C}+\theta_{C}\sin\theta_{C}-1\right)\left(\cot\frac{\theta_{C}}{2}\csc^{2}\frac{\theta_{C}}{2}\right)$.

For segment BC, the rotational equation of motion about point B is

(48) $$2F_{C}R\sin^{2}\frac{\theta_{C}}{2}-2M_{p}=\frac{dJ_{BC}}{dt}=\frac{dJ_{BC}}{d\dot{\alpha }}\ddot{\alpha }+\frac{dJ_{BC}}{d\theta_{C}}{\dot{\theta }}_{C}.$$

Substitution of Equation (16) into (48) gives

(49) $$\begin{array}{l} 2F_{C}R\sin^{2}\frac{\theta_{C}}{2}-2M_{p}\\ =2\rho R^{3}\left[\cot^{2}\frac{\theta_{C}}{2}\left(\theta_{C}-\sin\theta_{C}\right)-\sin\theta_{C}\right]\ddot{\alpha }\\ +2\rho R^{3}\left[\left(-\cot\frac{\theta_{C}}{2}\csc^{2}\frac{\theta_{C}}{2}\right)\left(\theta_{C}-\sin\theta_{C}\right)+\sin\theta_{C}-\cos\theta_{C}\right]\dot{\alpha }{\dot{\theta }}_{C} \end{array}.$$

For segment AC, the rotational equation of motion about point A is

(50) $$2F_{C}R\cos^{2}\frac{\theta_{C}}{2}-M_{p}=\frac{d}{dt}J_{AC}=\frac{dJ_{AC}}{d\dot{\alpha }}\ddot{\alpha }+\frac{dJ_{AC}}{d\theta_{C}}{\dot{\theta }}_{C}.$$

By substituting Equation (19) into Equation (50), one gets

(51) $$2F_{C}R\cos^{2}\frac{\theta_{C}}{2}-M_{p}=2\rho R^{3}\left(\pi -\theta_{C}-\sin\theta_{C}\right)\ddot{\alpha }+2\rho R^{3}\left(-1-\cos\theta_{C}\right)\dot{\alpha }{\dot{\theta }}_{C}.$$

Given the initial conditions at $t=t_{1}$ of phase II, which are calculated in phase I, Equations (47), (49), and (51) can be solved utilizing the Runge–Kutta method. The angular coordinate $\theta \left(t\right)$ of traveling hinge C, the angular velocity $\dot{\alpha }\left(t\right)$, and the angular displacement $\alpha \left(t\right)$ of segment BC are obtained. In phase II, the kinetic energy of the circular ring is dissipated by the traveling hinge C and the stationary hinge B. When $t=t_{2}$, the circular ring change over into a new stationary-hinge mechanism at a certain point and phase II ends. The ultimate angular coordinate of traveling hinge C in phase II is $\theta_{2}$. The angular velocity and angular displacement of segment AC at $t=t_{2}$ are ${\dot{\alpha }}_{2}$ and $\alpha_{2}$, respectively.

### 3.3. Phase III

When $t=t_{2}$, the traveling hinge C stops at the position of $\theta =\theta_{2}$. In the newly formed stationary-hinge mechanism, the kinetic energy of the circular ring is dissipated by the stationary hinges B and C. The angular velocity of the segments decreases with time uniformly. By substituting $\theta_{C}=\theta_{2}$ into Equations (18) and (21), the angular acceleration ${\ddot{\alpha }}_{2}$ is obtained. The angular velocity and angular displacement of segment AC in phase III are

(52) $$\dot{\alpha }\left(t\right)={\dot{\alpha }}_{2}+{\ddot{\alpha }}_{2}\left(t-t_{1}\right),$$

and

(53) $$\alpha \left(t\right)=\alpha_{2}+{\dot{\alpha }}_{2}\left(t-t_{2}\right)+\frac{1}{2}{\ddot{\alpha }}_{2}{\left(t-t_{2}\right)}^{2}.$$

As the kinetic energy is completely dissipated at $t=t_{3}$, phase III ends. From Equation (52) one gets

(54) $$t_{3}=t_{2}-\frac{{\dot{\alpha }}_{2}}{{\ddot{\alpha }}_{2}}.$$

## 4. Numerical Results

### 4.1. Step-Loading Response

**Figure 3:**
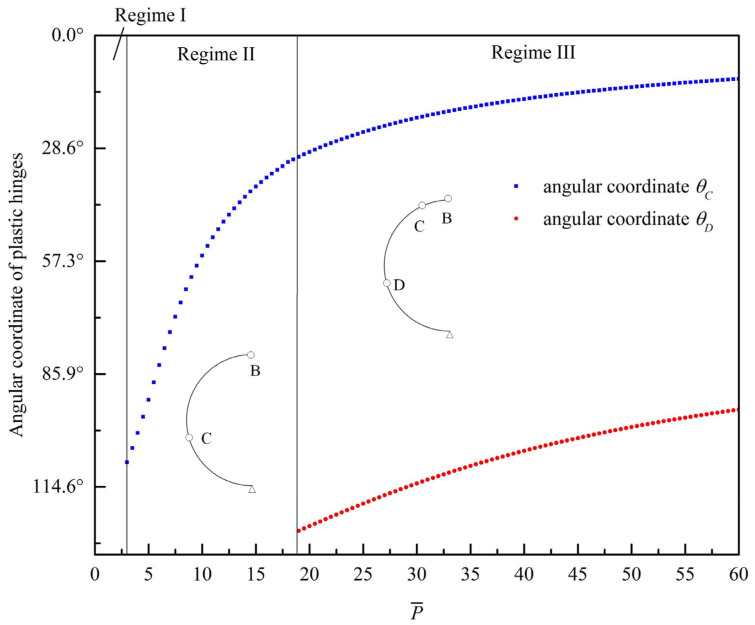
Relation between hinge positions and $\bar{P}$.

**Figure 4:**
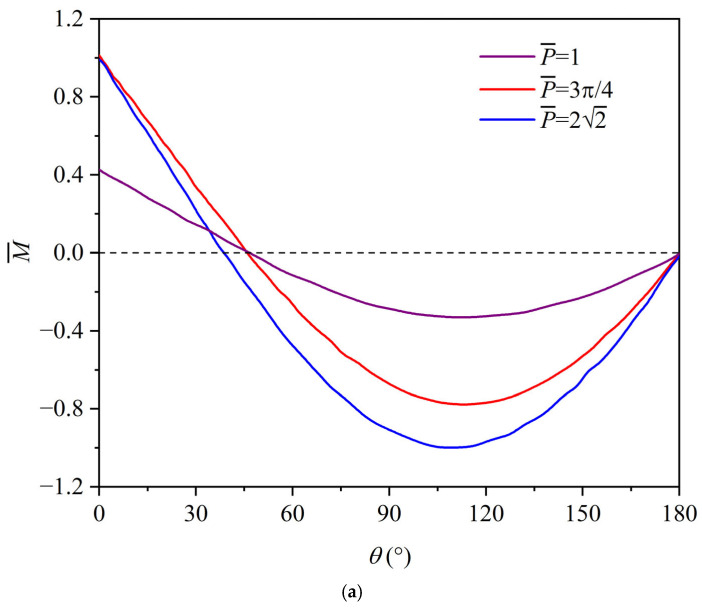

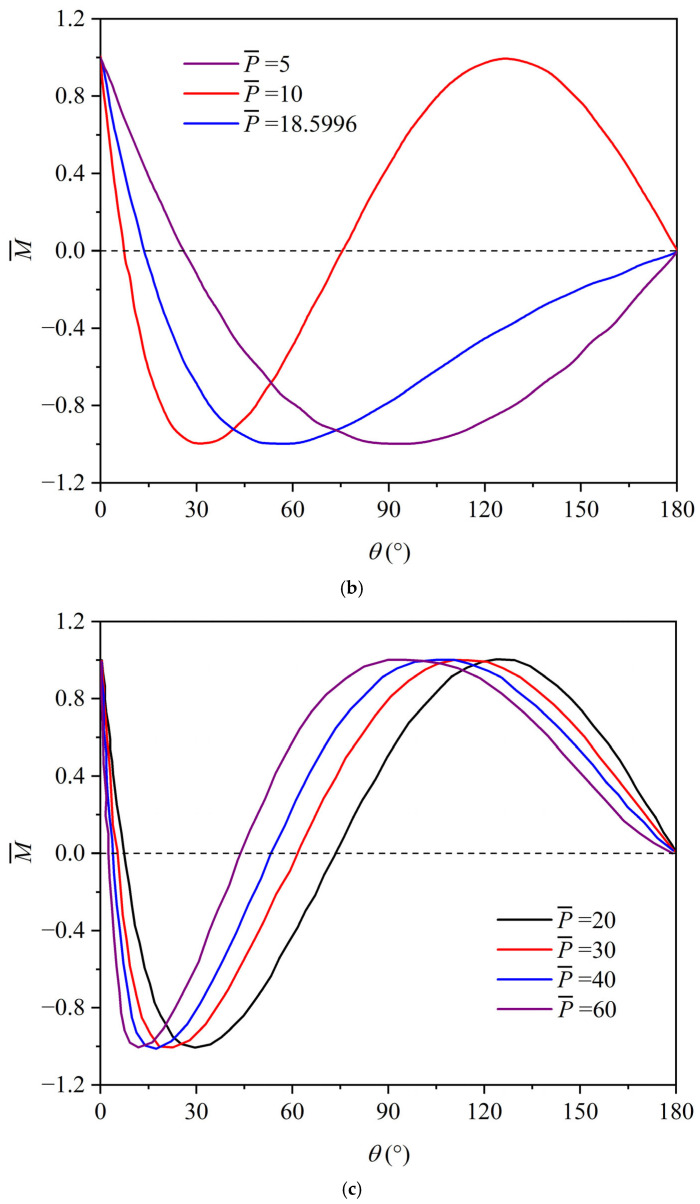
Non-dimensional bending moment distribution in the circular ring: (**a**) regime I, (**b**) regime II, (**c**) regime III. Dashed line represents the zero reference line.

Once the step loading is applied to the top of the circular ring, the rotation mechanism of the ring remains unchanged, as shown in the labels I, II, and III of Figure 2. Based on the rigid-perfectly plastic model in Section 2, one can get the relation between the plastic hinge locations $\theta_{C}$, $\theta_{D}$ and the non-dimensional step loading $\bar{P}$, as shown in Figure 3. The blue and red dots in Figure 3 represent hinges C and D in Figure 2, respectively. Considering the symmetry of the whole circular ring, hinges C and D are numbered as 2(3) and 4(5), respectively. The first hinge B is always located at the loading position and thus is omitted in Figure 3. The model is capable of calculating the bending moment distribution across the entire circular ring for a given load. The physical transitions between regimes are directly driven by the local bending moments reaching the plastic yield limit ($\bar{M}=1$). The distribution of the non-dimensional bending moment $\bar{M}=M/M_{p}$ in the circular ring is shown in Figure 4.

Pre-yield and transition to regime I (one-hinge): As depicted in Figure 4a, when $\bar{P}=1$, the bending moment is maximal at approximately θ=112° but remains less than 1, indicating the entire ring is still in the elastic state. Calculations show that as $\bar{P}<3\pi /4$, the whole circular ring is fully elastic. As $\bar{P}$ increases to between 3π/4 and $2\sqrt{2}$, only point B (θ=0°) enters the plastic state. As the applied load continues to increase, the bending moment across the rest of the ring increases while the bending moment at point B remains constant at the yield limit.

Transition to regime II (three-hinge): When the load increases to $\bar{P}=2\sqrt{2}$, the bending moment at θ=109° reaches 1. This physical threshold dictates the formation of plastic hinge C, which regime I ends. For $2\sqrt{2}\leq \bar{P}<18.5996$, the circular ring lies in regime II, characterized by three plastic hinges in the whole ring. As shown in Figure 4b, the yield criterion is satisfied. As the external load continues to increase (e.g., from $\bar{P}=5$ to 10), the plastic hinge C approaches point B (moving from θ=93° to 55°), and the bending moment in segment AC enhances accordingly with the movement of hinge C.

Transition to regime III (five-hinge): When $\bar{P}$ reaches 18.5996, the bending moments at the θ=0°, 31°, and 126° positions reach 1. This causes a new plastic hinge D to form. For $\bar{P}>18.5996$, the circular ring lies in regime III, resulting in five plastic hinges across the whole circular ring.

As discussed in the revised manuscript, the most important dimensionless parameter governing the bifurcation of deformation modes is $\bar{P}$. For each deformation regime, it is found that, as $\bar{P}$ increases, the newly formed plastic hinges move progressively closer to the point of external loading. This trend suggests that increasing load magnitude leads to a stronger localization of plastic deformation in the vicinity of the applied load. Such a feature is analogous to the response of a straight uniform beam subjected to a tip step load, where the plastic deformation region tends to concentrate toward the impact point [53,54]. This similarity provides further support for the validity and physical consistency of the present theoretical results. From Figure 4c, it is seen that as the non-dimensional load is less than 60, the yield criterion is still satisfied, which indicates that this five-hinge mode is still effective.

### 4.2. Validation of the Deformation Mechanism

In this section, the commercial finite element software package ABAQUS 2022 (Dassault Systemes Simulia Corp., Providence, RI, USA) is used to verify the deformation mechanisms under step loading proposed in the present rigid-plastic model. It is specifically designed to predict the incipient deformation mechanisms (i.e., the number and initial locations of plastic hinges) and the initial collapse load. The geometric parameters of the circular ring are set as *R* = 100 mm, *h* = 2 mm, and *b* = 5 mm, respectively. Due to symmetry, only half of the circular ring is shown. The circular ring is meshed by using the four-node bilinear plane stress quadrilateral element with reduced integration and hourglass control (CPS4R) and discretized with 2094 elements. The yield stress is set as 300 MPa. Due to the neglect of elastic deformation and strain hardening, the RPP model naturally lacks the resolution to accurately predict the continuous displacement history or energy dissipation (which depends on large-scale plastic flow and geometry change). Furthermore, since the model employs a step-loading or impulsive-loading idealization, the “hinge formation time” in the theory is often treated as instantaneous.

**Figure 5:**
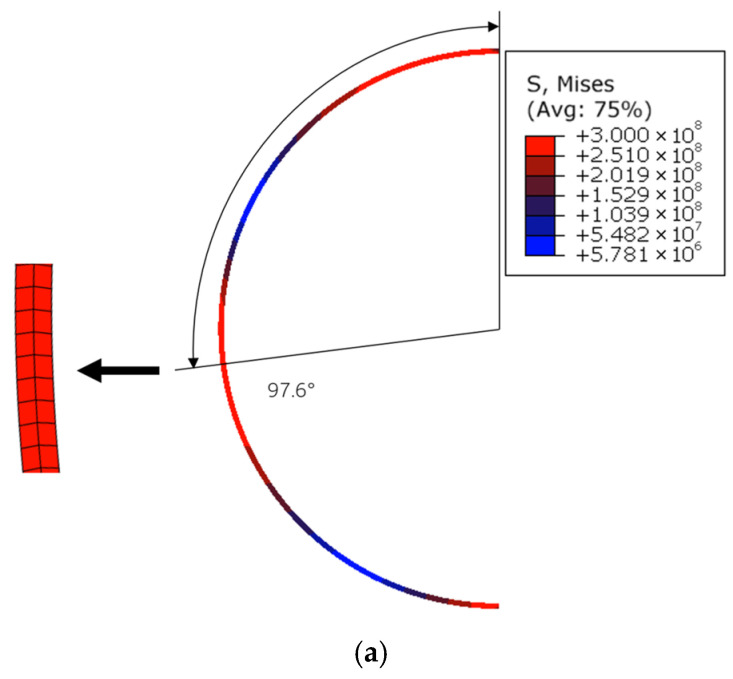

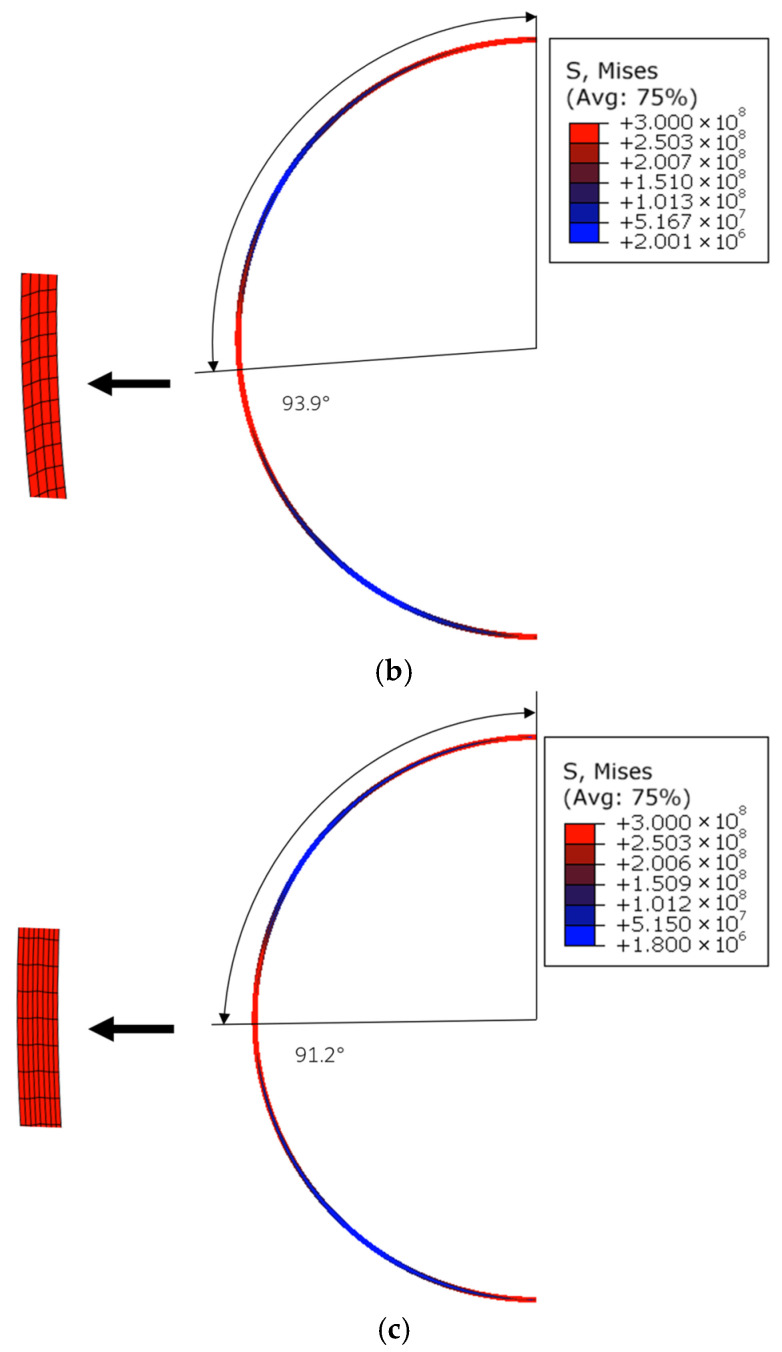
Mesh sensitivity analysis of finite element methods for $\bar{P}=5$ (stress unit: MPa; element type: CPS4R). (**a**) Coarse mesh. (**b**) Baseline mesh. (**c**) Fine mesh.

**Figure 6:**
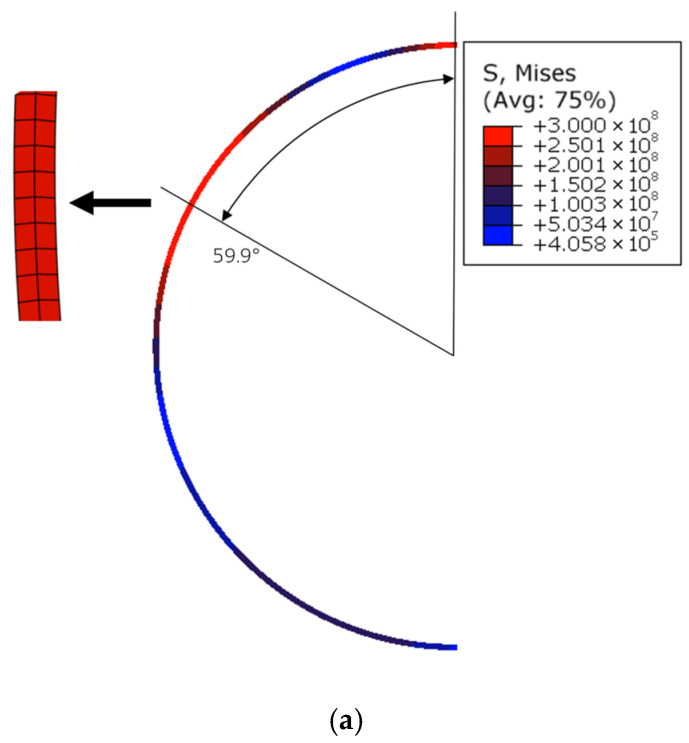

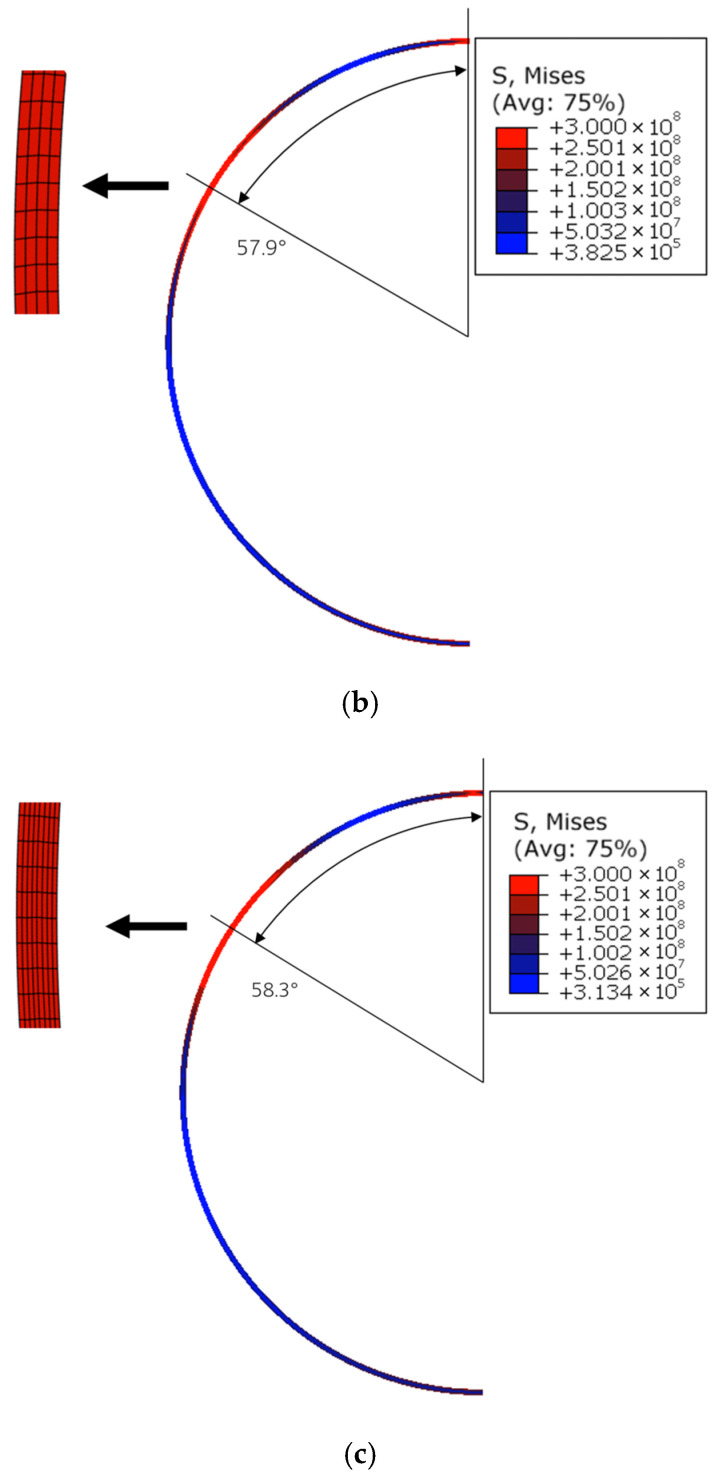
Mesh sensitivity analysis of finite element methods for $\bar{P}=10$ (stress unit: MPa; element type: C3D8R). (**a**) Coarse mesh. (**b**) Baseline mesh. (**c**) Fine mesh.

To strengthen the finite element validation, we have carried out sensitivity analyses with respect to mesh density and element type. Specifically, it was discretized using two, four, and eight elements through the thickness of the ring, which represent coarse mesh, baseline mesh, and fine mesh, respectively. In addition, both plane-stress elements (CPS4R) and solid elements (C3D8) were examined, as shown in Figure 5 and Figure 6, respectively. We evaluated the plastic hinge locations, which are the primary outputs of our model. The relative errors between the theoretical predictions and the FEA results are shown in Table 1. The results show that discretization with four elements (baseline mesh) through the thickness already provides sufficient accuracy and balances the computational expense. The comparison also demonstrates that the CPS4R elements adopted in the study are appropriate for the present thin-walled ring problem. Based on the above results, it can be concluded that the finite element model adopted in this study provides sufficient accuracy.

**Figure 7:**
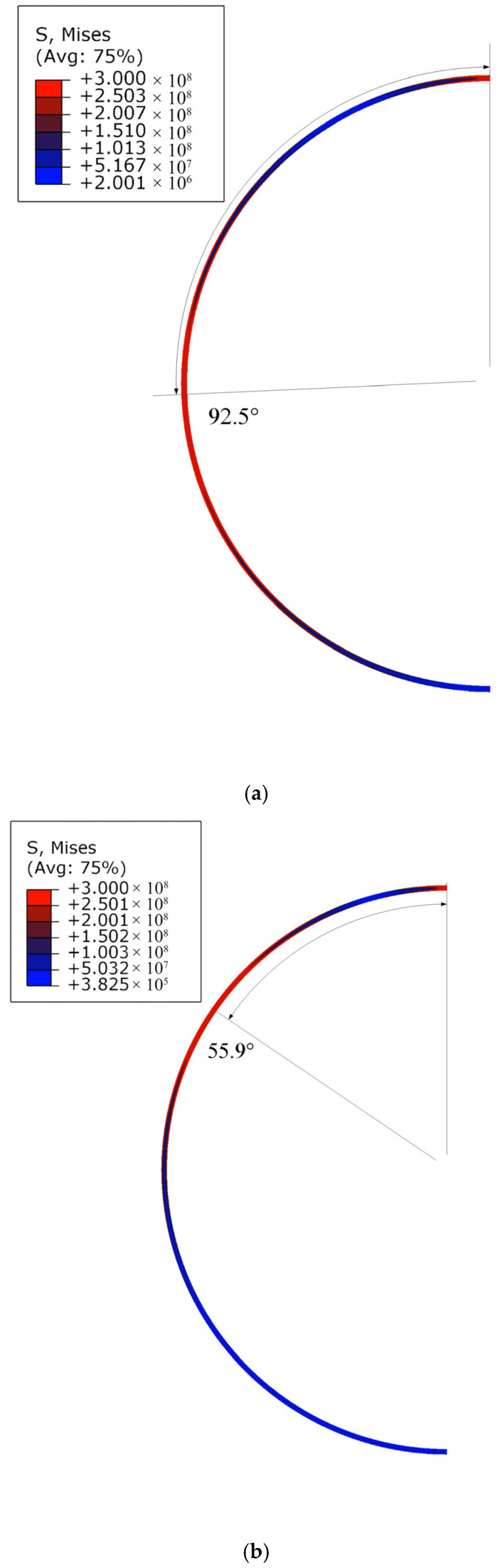

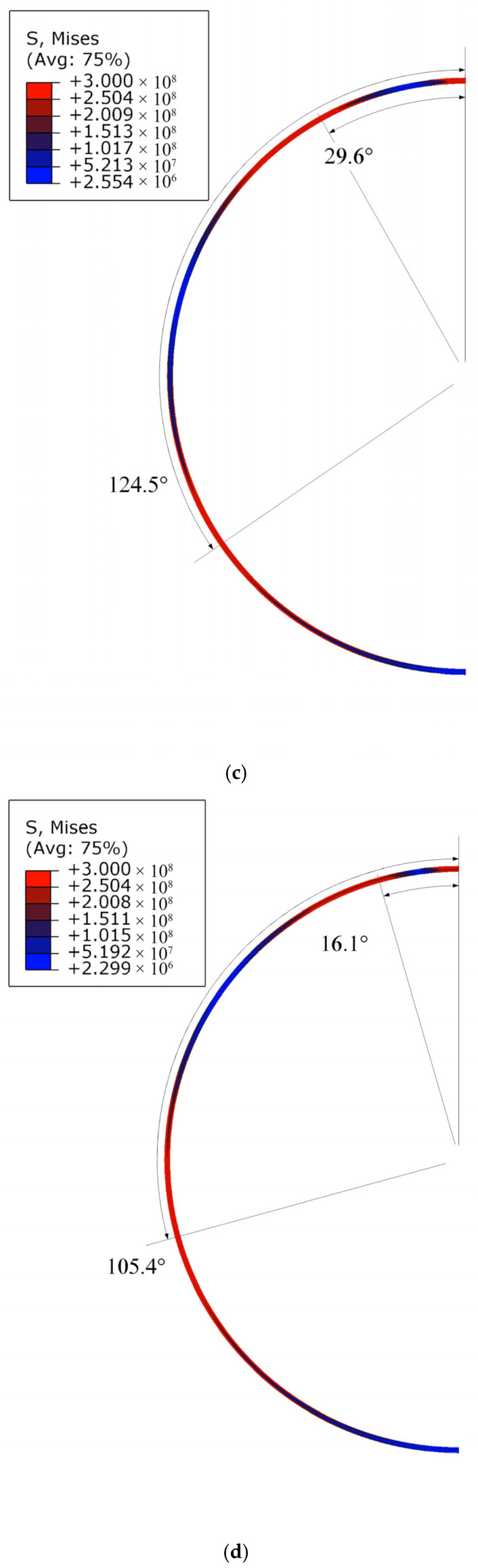

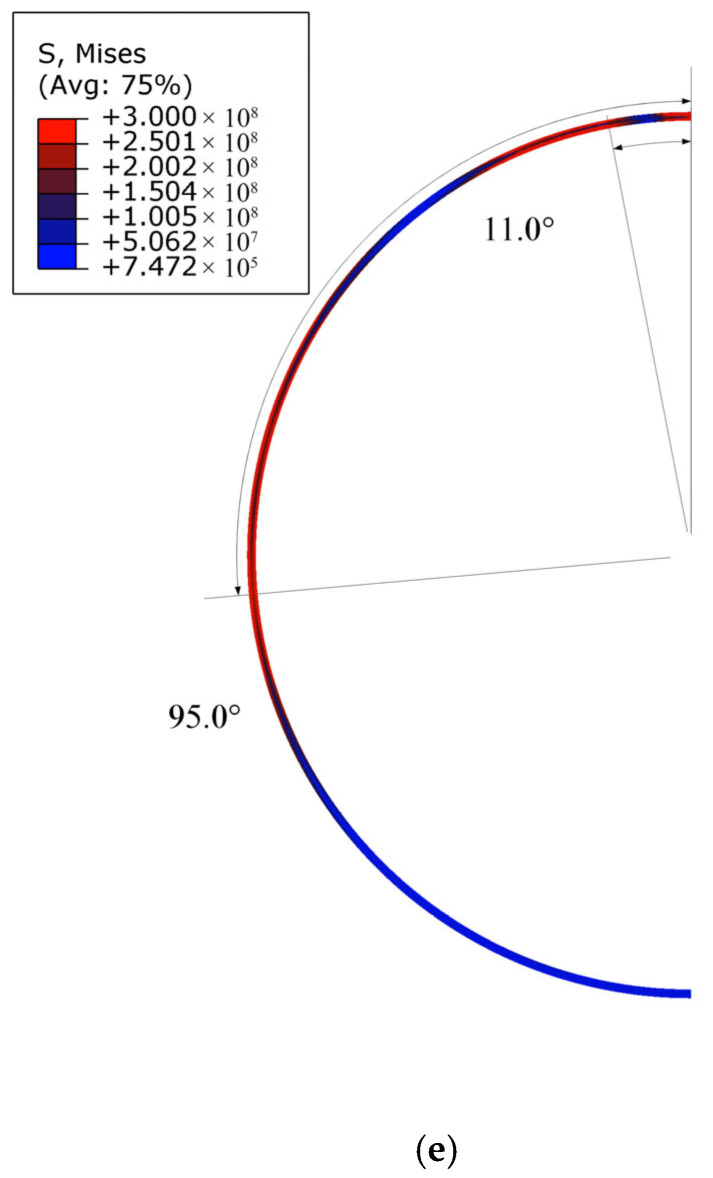
Comparison between theoretical predictions and finite element results for step loading: (**a**) $\bar{P}=5$, (**b**) $\bar{P}=10$, (**c**) $\bar{P}=20$, (**d**) $\bar{P}=40$, and (**e**) $\bar{P}=60$.

In Figure 7, the von Mises stress contours are given for $\bar{P}=5,\;10,\;20,\;40,\;60$, respectively. The notations 92.5°, 55.9°, 29.6°, 16.1°, and 11.0° represent the theoretical predictions of the positions of plastic hinge C, and 124.5°, 105.4°, and 95.0° are the locations of plastic hinge D. The theoretical predictions of the deformation mechanisms are in good agreement with those predicted by the finite element simulation as shown in Figure 7. For $\bar{P}=5$ and $\bar{P}=10$, the circular ring deforms with the three-hinge mode. For $\bar{P}=20$, $\bar{P}=40$, and $\bar{P}=60$, the circular ring deforms with the five-hinge mode.

Comparison of the stress contours shows that the plastic hinges move towards the loading position as the load increases, which is accordant with the theoretical predictions. In actual structures, plastic deformation takes place over a region instead of at a point [9], which means that the hinges have a certain length. It is coincident with the results of the finite element analysis shown in Figure 7. When the load is small, the plastic hinges predicted by the rigid plastic model are located at the central zone of the plastic region in the von Mises stress contour. As the magnitude of the load gets larger, the theoretical result deviates gradually from the central zone of the plastic region predicted by the finite element analysis. As $\bar{P}$ increases, the predicted hinge position exhibits a slight shift away from the center of the plastic region. Nevertheless, it consistently falls within the hinge location range obtained from finite element simulations. In the presented model, the shear and axial deformations of the circular ring are neglected.

We have further examined the roles of axial force and shear force based on the finite element results. In the example, at the onset of plastic hinge formation, $M/M_{p}=1$. The tensile yield stress $\sigma_{s}$ is set as 300 MPa. According to the von Mises criterion, the shear yield stress can be estimated as $\tau_{s}=\sigma_{s}/\sqrt{3}$ [52]. The axial and shear force under different step loadings were calculated, as shown in Table 2.

For $\bar{P}=5$, 10, 20, 40, and 60, the contribution of axial and shear force (measured by $N/N_{p}+T/T_{p}$) is approximately 0.38%, 0.47%, 0.71%, 3.52%, and 15.29%, respectively. These results indicate that while the model precisely predicts the number of hinges across a wide range, the error in hinge locations becomes noticeable when $\bar{P}=60$ due to the increasing influence of the neglected axial and shear forces. Thus, the present analytical predictions are most reliable for $\bar{P}<40$, providing clear guidance for its engineering application.

Under low or moderate impact loads, the predicted number and locations of the plastic hinges agree with previous experimental observations [14,55,56]. With increasing impact magnitude, the deformation mechanism gradually evolves from a three-hinge mode to a five-hinge mode, which is also consistent with the dynamic plastic deformation modes reported by Stronge et al. [47] for suddenly loaded curved beams and pinned-supported rings. In addition, our model reveals that as the load magnitude increases, the newly appeared plastic hinges move progressively closer to the externally applied load. This behavior is analogous to a straight uniform beam subjected to tip step loading, where the plastic deformation zone localizes toward the impact point, as proven by experimental and theoretical analyses [53,54].

### 4.3. Pulse-Loading Response

Based on the theoretical model of the circular ring, the dynamic responses of the circular ring under rectangular pulse loading are obtained. Considering the constraint $2\sqrt{2}\leq {\bar{P}}_{0}<18.5996$ of regime II in Figure 2, the magnitudes of the load are set as ${\bar{P}}_{0}=5,\;10,\;15$, respectively. The duration $t_{1}$ is set as 1 ms.

**Figure 8:**
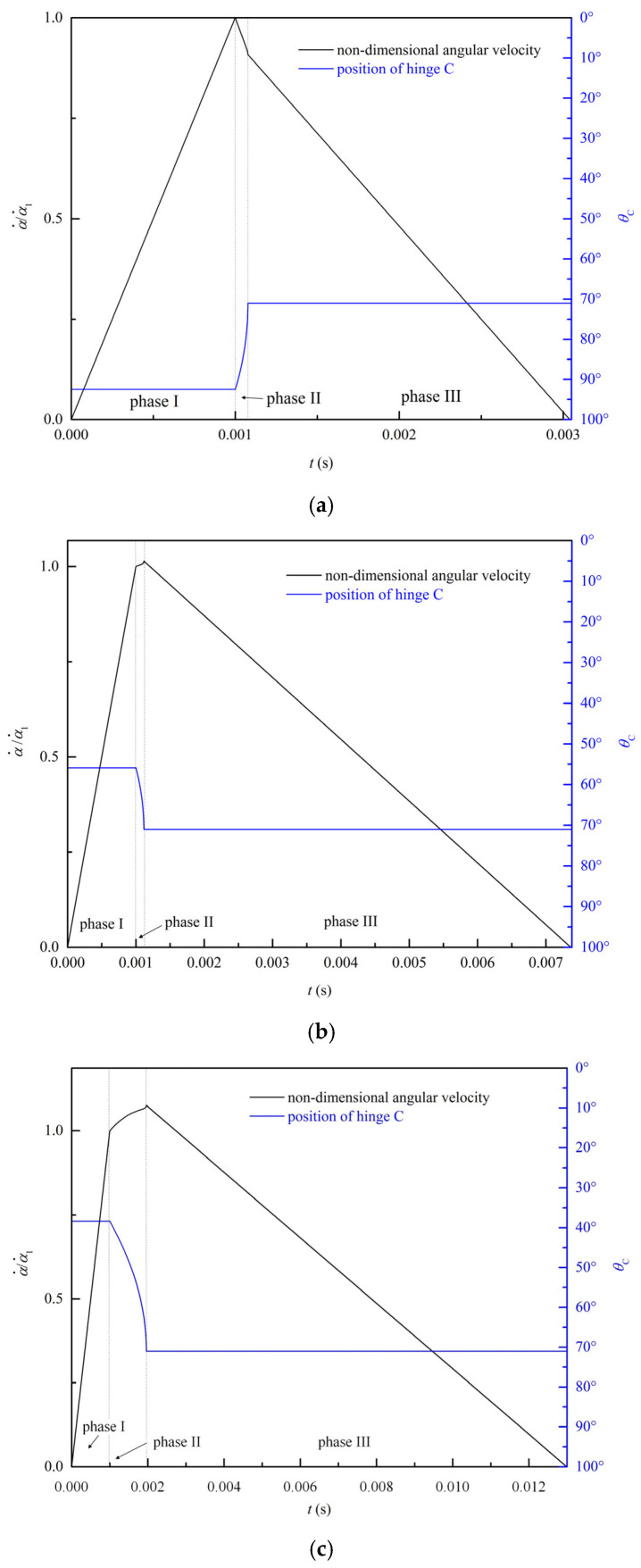
Variation of the non-dimensional angular velocity of segment AC and the angular coordinate of hinge C: (**a**) ${\bar{P}}_{0}=5$, (**b**) ${\bar{P}}_{0}=10$, and (**c**) ${\bar{P}}_{0}=15$.

As stated in Section 3, the first hinge B is located at the loading point and remains unchanged. The second hinge C is located at different positions depending on the magnitude of the pulse. Meanwhile, the state of hinge C and the angular velocities of the segments also change over time. Utilizing the angular velocity ${\dot{\alpha }}_{1}$ of the segment AC at the time when the pulse is removed, the non-dimensional angular velocity is defined as $\dot{\alpha }/{\dot{\alpha }}_{1}$. The variations of the non-dimensional angular velocity of segment AC and the angular coordinate of hinge C over time are depicted in Figure 8. As shown in Figure 8a, when the load ${\bar{P}}_{0}=5$ is applied to the ring, the stationary hinge C forms at θ=92.49°. In phase I, the angular velocity of segment AC increases linearly with time until $t=t_{1}$. In phase II, when the pulse *P*_0_ is removed at $t=t_{1}$, the plastic hinge C becomes a traveling hinge and moves close to the loading position B along the circular ring. The angular velocity of segment AC decreases gradually. As the traveling hinge C stops at the position of θ=71.03° at $t_{2}=1.1\;\mathrm{ms}$, phase II ends. In phase III, the plastic hinge C stays at θ=71.03°, and the angular velocity of the segments decrease linearly with time until the rest of the kinetic energy is completely dissipated by the stationary hinges.

The dynamic responses of the circular ring for ${\bar{P}}_{0}=10$ and ${\bar{P}}_{0}=15$ are similar to that of ${\bar{P}}_{0}=5$, as shown in Figure 8b and Figure 8c, respectively. In phase I, the stationary hinges form at θ=55.84° and θ=38.37° for ${\bar{P}}_{0}=10$ and ${\bar{P}}_{0}=15$, respectively. In phase II, both of the two hinges move towards the position of θ=71.03°. In phase III, these two plastic hinges remain stationary at θ=71.03° until the response ends. It can be concluded that the movement of hinge C is similar to that of clamped beams under rectangular pulse loading as depicted in the previous study [46]. A stationary hinge forms at a certain position once the pulse is loaded and travels to another stationary position after the pulse is removed.

## 5. Discussion

In this section, we discussed the advantages and disadvantages of the theoretical model. Although the rigid-perfectly plastic material model (RPP model) has certain limitations, it still provides a distinct advantage in revealing the initial stages of deformation. It offers a mathematically concise and physically clear framework to determine the precise relation between the magnitude of the applied load and the number and location of plastic hinges. This approach establishes a foundation before introducing highly complex non-linearities.

Based on the theoretical framework, we can establish a fundamental scaling relationship between the applied impulse, the ring geometry, and the total plastic work dissipated. Under an idealized short-duration impulsive load I, the initial kinetic energy $E_{in}$ imparted to the structure can be estimated as [52]

(55) $$E_{\mathrm{in}}\propto \frac{I^{2}}{2m}=\frac{I^{2}}{4\pi \rho Rbh}$$

where m is the mass of the ring, and ρ is the material density.

To arrest the impact, this kinetic energy must be entirely dissipated through plastic work $W_{p}$ at the plastic hinges. The plastic work is the product of the fully plastic bending moment $M_{p}$ and the equivalent cumulative rotation angle $\Delta \theta_{eq}$ at the hinges [52]:

(56) $$W_{p}\propto M_{p}\Delta \theta_{eq}=\left(\frac{\sigma_{0}bh^{2}}{4}\right)\Delta \theta_{eq}$$

By equating $E_{in}$ and $W_{p}$, the scaling relationship for the required rotational deformation is obtained:

(57) $$\theta_{eq}\propto \frac{I^{2}}{\pi \rho \sigma_{0}b^{2}R^{4}}{\left(\frac{R}{h}\right)}^{3}$$

This scaling law clearly reveals the profound influence of the radius-to-thickness ratio R/h on the structural crashworthiness. For a given material (ρ, $\sigma_{0}$), radius (R) and impulse (I), the total rotational angle on the hinges is proportional to the cube of the R/h ratio.

Furthermore, the evolution of the deformation mechanisms has an effect on the efficiency of energy absorption. For example, in the lower-energy regime II, energy is absorbed at only three locations. However, as the impact magnitude increases and the ring transitions to the five-hinge mode (Regime III), the number of active plastic hinges increases, and energy is dissipated over a larger volume of the ring. This spatial distribution of plastic work enhances the energy dissipation efficiency of the circular ring structure.

Under high-rate impulsive loading, actual materials typically involve strain hardening, strain-rate sensitivity, geometric imperfections, and finite strain effects. Firstly, incorporating strain hardening would increase the material’s plastic yield bending moment ($M_{P}$) as deformation progresses. Consequently, this would raise the critical load thresholds required to initiate subsequent plastic hinges. However, it would not qualitatively alter the fundamental evolution mechanism. The number of hinges would still increase, and their newly formed positions would continue to move closer to the loading point as the load magnitude increases.

Similarly, if we consider the strain-rate sensitivity (e.g., as described by the Cowper–Symonds model), the dynamic yield stress is given by [57]

(58) $$\sigma_{d}=\sigma_{y}\left[1+{\left(\dot{\varepsilon }/D\right)}^{1/q}\right]$$

where $\sigma_{y}$ is the static yield stress, $\dot{\varepsilon }$ is the equivalent plastic strain rate, and D and q are material constants. Consequently, the dynamic fully plastic bending moment $M_{d}$ becomes a function of the local curvature rate $\dot{\kappa }$, yielding $M_{d}\left(\dot{\kappa }\right)>M_{p}$. The primary implication of this rate dependence is an elevation of the critical threshold load required to initiate a stationary plastic hinge. However, because the spatial gradient of the bending-moment field is largely dictated by the load distribution and structural boundary conditions in the incipient stage, the predicted number and qualitative distribution patterns of the stationary hinges remain consistent with the RPP model.

In the context of the present model, the effect of geometric imperfections is highly dependent on their size. For small-scale imperfections, the initial distribution of plastic hinges is predominantly governed by the external load and the primary structural topology, meaning the results of the present analysis remain robust during the incipient phase of loading. However, large-scale imperfections can fundamentally alter the structural geometry and boundary conditions from the initial state, leading to a redistribution of the bending-moment field:

(59) $$M\left(x,t\right)=f\left(\mathrm{Load},\;\mathrm{Geometry}+\Delta \mathrm{Imperfection}\right)$$

where ΔImperfection represents the deviation from the ideal configuration. Furthermore, even minor imperfections may lead to significant localized instabilities when the structure transitions into the large-deflection regime, where geometric nonlinearities become dominant. Due to the high complexity of modeling these stochastic imperfection fields and their coupling with rate-dependent plasticity, this study focuses on the idealized structural configuration to provide a clear physical solution. A systematic investigation into the sensitivity of hinge evolution to geometric imperfections is identified as a key objective for our future research.

Furthermore, finite strain effects would introduce geometric nonlinearities and axial-shear-bending coupling, thereby changing the moment arms of the applied forces and internal stresses. In summary, introducing rate-dependent plasticity or finite strain effects into the present model would significantly increase mathematical complexity, making analytical solutions extremely difficult to obtain.

Finally, we discussed the potential effects of the non-ideal boundary conditions and complex loadings. If the supports were clamped or partially rotationally constrained, the boundary conditions in our derivation would change (e.g., introducing a non-zero bending moment at the support). While the fundamental governing equations of our rigid-plastic framework would remain applicable, the structural response would change. Specifically, we would expect stationary plastic hinges to form earlier at or near the constrained supports to dissipate energy, altering the threshold loads for transition between the one-, three-, and five-hinge regimes.

The current analytical model relies on the symmetric deformation of the circular ring to simplify the degrees of freedom. An eccentric impact would break this symmetry. While the underlying rigid-plastic theory still holds, extending the current framework to asymmetric loading would require abandoning the symmetry assumption, which would significantly increase the degrees of freedom. Tracking the independent traveling hinges on both sides of the loading point would drastically increase the mathematical and physical complexity, likely requiring semi-analytical or numerical methods to solve the differential equations. Under a point load, the maximum bending moment is sharply localized at the loading point, leading to the immediate formation of the first plastic hinge. If the load were distributed over a region, the bending-moment peak would flatten. Consequently, rather than forming a distinct point-like stationary hinge, the ring might develop a wider plastic deformation zone, or the primary hinges might form at the edges of the distributed load area. As the magnitude of the distributed load continues to increase, the bending moment at other locations on the circular ring will also eventually reach the fully plastic yield limit, which will lead to the sequential formation of additional plastic hinges across the structure. This underlying physical mechanism remains fundamentally consistent with our findings for the concentrated point load.

As mentioned above, the current model relies on rigid-perfectly plastic material behavior and small deflection. Furthermore, it neglects the effects of axial and shear forces on the yielding criterion, which we found creates noticeable discrepancies under extremely high loads. Therefore, the primary limitation in applying this analytical solution to real structural applications is that it is strictly valid only for predicting the incipient numbers and locations of plastic hinges. In real cases, post-stage crushing involving large deformations, strain hardening, and strain-rate sensitivities, finite element analysis becomes necessary.

## 6. Conclusions

In this study, we proposed an analytical model to investigate the incipient plastic response of simply supported circular rings subjected to impact loads. The rigid-plastic theory is employed, and the deformation mode of the circular ring under different magnitudes of the step loading is examined. It is found that with an increase in step loading, the deformation mode of the circular ring changes. The theoretical solutions for one-, three-, and five-hinge modes are derived as the step loading increases. The theoretical results are validated by using finite element analyses. It is found that the deformation mechanisms predicted by the proposed theoretical model are in good agreement with the numerical results. In these modes, the plastic hinges move towards the loading position as the magnitude of the load increases. The dynamic responses of the circular ring under rectangular pulse loading are further investigated for different load magnitudes. Both stationary and traveling hinges may exist in the circular ring. For different load magnitudes, the plastic hinges form at different positions in phase I. As the load is removed at the beginning of phase II, the hinges start to move along the circular ring and travel to the same position finally. In phase III, the hinges remain stationary and the segments decelerate gradually until the kinetic energy is completely dissipated by the plastic hinges.

It should be noted that the proposed model relies on rigid-perfectly plastic material behavior and linear kinematics, which restricts its validity to the initial deformation stage. Specifically, the neglect of axial and shear force effects leads to discrepancies under extremely high loads. Despite these restrictions, the model successfully clarifies the relation between load magnitude and the evolution of plastic hinges, offering essential theoretical insights for further studies on large-deformation behaviors of thin-walled structures under more complicated impact loads and boundary constraints.

## Figures and Tables

**Table 1:** Mesh sensitivity analysis of FEA results.

Step Loading	Theoretical	CM	BM	FM	Relative Errors with BM
$\bar{P}=5$	92.5°	97.6°	93.9°	91.2°	1.5S%
$\bar{P}=10$	55.9°	59.9°	57.9°	58.3°	3.6%

Note: CM represents coarse mesh, BM represents baseline mesh, and FM represents fine mesh.

**Table 2:** The contribution of axial and shear force.

Step Loading	$N/N_{p}$	$T/T_{p}$	$N/N_{p}+T/T_{p}$
5	0.16%	0.22%	0.38%
10	0.23%	0.24%	0.47%
20	0.39%	0.32%	0.71%
40	1.56%	1.96%	3.52%
60	5.58%	9.71%	15.29%

Note: $N_{p}=\sigma_{s}bh$ and $T_{p}=\tau_{s}bh$.

## Data Availability

The original contributions presented in this study are included in the article. Further inquiries can be directed to the corresponding author.
